# Compressive optic neuropathy due to posterior ethmoid mucocele

**DOI:** 10.1186/s12886-023-03168-w

**Published:** 2023-10-23

**Authors:** Song-A Che, Yong Woo Lee, Yung-Ju Yoo

**Affiliations:** 1 Department of Ophthalmology, Kangwon National University Hospital, Chuncheon, South Korea; 2 Department of Ophthalmology, Kangwon National University School of Medicine, Chuncheon, South Korea

**Keywords:** Acute visual loss, Compressive optic neuropathy, Mucocele, Posterior ethmoid cell

## Abstract

Mucoceles are cystic formations characterized by the presence of mucus-secreting epithelial cells, which enlarge when the excretory duct becomes obstructed. Posterior ethmoidal mucoceles are rare conditions that can lead to severe ocular complications requiring immediate intervention. The close anatomical proximity of posterior ethmoidal mucoceles to the optic nerve underscores their significance. In this case report, we present a case of rapidly progressing compressive optic neuropathy secondary to a posterior ethmoidal mucocele. A previously healthy forty-six-year-old woman presented with sudden visual loss in her left eye, preceded by left-sided headache and periorbital pain. Clinical examination and imaging studies revealed an oval-shaped mass within the posterior ethmoid cell compressing the left optic nerve. Emergency surgery was performed to alleviate optic nerve compression, which successfully relieved periocular pain. However, the patient’s visual acuity and visual field defect remained unchanged postoperatively. Thinning of the ganglion cell layer in the macula region was observed during follow-up examinations. The role of corticosteroids and antibiotics in visual rehabilitation and the impact of delayed surgical decompression on visual outcome remain subjects of debate. Additional cases of mucocele-associated optic neuropathy should be published and analyzed to establish optimal treatment approaches.

## Background

Mucoceles are cystic formations characterized by the presence of mucus-secreting epithelial cells [1]. When the excretory duct becomes obstructed, mucus accumulates within the cyst, leading to its enlargement [1]. Posterior ethmoidal mucoceles are rare conditions that can potentially give rise to severe ocular complications requiring immediate intervention. The significance of posterior ethmoidal mucoceles lies in their close anatomical proximity to the optic nerve [2]. Unilateral retrobulbar optic neuropathy can arise from various causes, often posing challenges in accurate diagnosis. In this report, we present a case involving a patient who developed optic nerve neuropathy as a result of a posterior ethmoidal mucocele. Herein, we elucidate the clinical presentation of a rapidly progressing compressive optic neuropathy secondary to a posterior ethmoidal mucocele.

## Case presentation

**Fig. 1:**
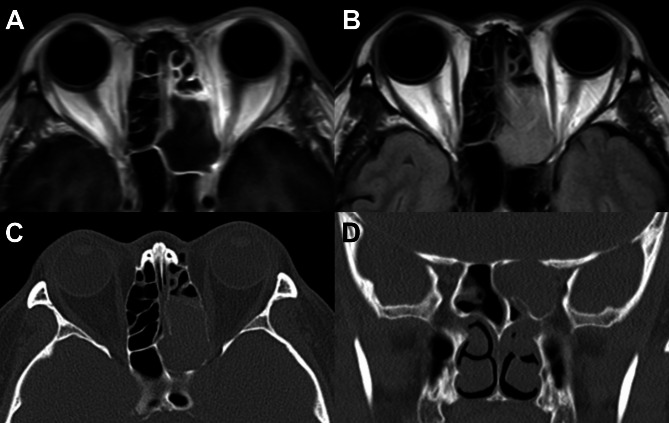
Preoperative Brain Magnetic Resonance Imaging (MRI) and Paranasal Sinus Computed Tomography (CT). (**A, B**) Preoperative brain MRI shows mass on the ethmoidal sinus, with intermediate signal on T1 image (A) and intermediate to low signal intensity on T2 FLAIR image (**B**). (**C, D**) Preoperative paranasal sinus CT. (**C**) Axial scan shows total opacification on the left ethmoidal cell. (**D**) Coronal scan shows prominent dehiscence of canal wall (arrowheads)

A previously healthy forty-six-year-old woman presented to the emergency department eight hours after experiencing a sudden visual loss in her left eye. She had a 5-day history of worsening left-sided headache, as well as a three-day history of progressive left-sided periorbital and facial pain. An ophthalmological examination was conducted at a primary clinic 10 h prior to her admission to the emergency room due to orbital pain, but no significant findings were observed. Best corrected visual acuity (BCVA) was 20/20 in the right eye and light perception positive in the left eye. Notably, a prominent relative afferent pupillary defect (RAPD) was observed in the left eye, accompanied by periocular pain. The slit-lamp examination revealed no remarkable findings, except for injection of the left nasal conjunctiva. Computed tomography (CT) scans demonstrated a heterogeneous oval-shaped mass located within the posterior ethmoid cell, extending towards the optic nerve at the infero-medial side of the optic canal, where bony dehiscence was observed (Fig. 1). Magnetic resonance imaging (MRI) revealed compression of the left optic nerve within the canal by an oval-shaped lesion originating from the posterior ethmoidal sinus, displaying low signal intensity on T1 and T2-weighted images (Fig. 1).

**Fig. 2:**
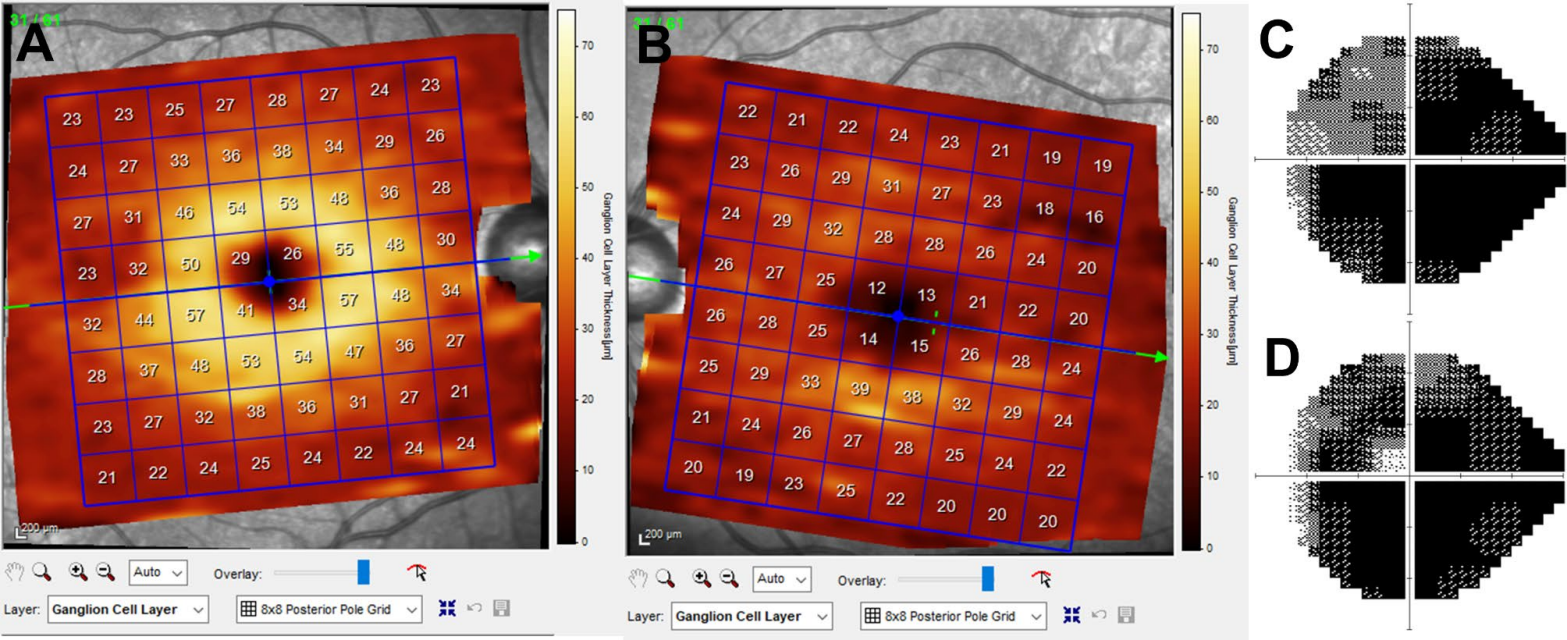
Postoperative Ophthalmic Findings. (**A**) optical coherence tomography of right eye (unaffected side). (**B**) optical coherence tomography of left eye (affected side) 3 weeks after surgery. Severe progression of ganglion cell layer thinning was noted. (**C**) Initial and (**D**) final visual field examination. Humphery visual field of the left eye showed significant constriction of the visual field

Emergency surgery was expeditiously undertaken based on comprehensive clinical and radiological assessments to alleviate optic nerve compression. The patient underwent emergency decompression operation by Endoscopic pansinus surgery(EPSS). The surgery was done by rhinology surgeon, ensuring the absence of iatrogenic optic nerve damage. To approach to posterior ethmoid cell, uncinectomy and partial conchotomy of middle turbinate was done. Anterior ethmoid cell was removed. Content of the posterior ethmoid mucocele was purulent and mucoid. Subsequent microbial culture analysis revealed the growth of Klebsiella aerogenes. Postoperatively, the patient received a regimen of intravenous antibiotics (Ceftriaxone 2000 mg I.V./twice a day) for a duration of three days. Remarkably, immediate alleviation of left periocular pain was unequivocally confirmed. Notably, the patient’s left eye achieved an improved best corrected visual acuity (BCVA) of hand motion (HM) on the day of the surgical intervention. Profound visual field constriction was evident during the left eye’s visual field examination, persisting unaltered even after a two-month follow-up period (Fig. 2). Noteworthily, the fundus exhibited normal findings postoperatively. The retinal nerve fiber layer (RNFL) thickness remained within normal limits during the postoperative day 2 and day 14 examinations (Fig. 2). Conversely, subsequent evaluations at one month and two months post-surgery unveiled discernible thinning of the ganglion cell layer within the macula region (Fig. 2).

## Discussion and conclusions

Posterior ethmoid mucocele has been associated with various detrimental effects, including retrobulbar optic neuropathy, cranial nerve palsies, and acute visual loss [1, 2]. The severity of optic neuropathy is influenced by both the size and location of the mucocele, as well as the direction of its expansion. Numerous ophthalmologic manifestations have been observed in such cases. The stretching of the dura mater and sinus mucosa can induce trigeminal nerve-mediated periorbital pain, a characteristic symptom that our patient experienced prior to the onset of visual loss [2].

Imaging techniques, such as computed tomography (CT) and magnetic resonance imaging (MRI), play a crucial role in the diagnosis of mucocele. CT scans can reveal thinning or erosion of the bony wall, as well as outline the expansile homogeneous mass. However, bone destruction is infrequent, and MRI surpasses CT in differentiating mucoceles from other soft tissue lesions, such as neoplasms.

Prompt surgical decompression serves as the primary treatment modality for optic neuropathy associated with sinus mucocele [3]. The impact of delaying surgical decompression on the ultimate visual outcome remains a topic of debate. Furthermore, the role of corticosteroids and antibiotics in visual rehabilitation remains uncertain [2]. A review of 24 published cases concerning optic neuropathy associated with posterior ethmoid cells revealed that the role of corticosteroids and antibiotics in visual rehabilitation remains unclear, and the degree of delay in surgical decompression did not significantly affect final visual acuity. Notably, the initial visual acuity was the sole prognostic factor indicating a poor outcome [2].

Optic neuropathy can occur through three distinct mechanisms. Firstly, the sudden enlargement of a mucocele directly compresses the optic canal, leading to bony dehiscence and subsequent damage to the optic nerve. Secondly, compression can impede the blood supply to the optic nerve. Thirdly, direct spread of mucocele-initiated inflammation into the optic nerve sheath, resulting in optic neuropathy [5]. Yumoto et al. reported a significantly higher rate of optic nerve dehiscence (86%) among patients with a mucocele of the posterior ethmoid or sphenoid sinus [4].

In our case, visual acuity and visual field worsened rapidly. And despite the successful implementation of urgent endoscopic optic nerve decompression surgery, the visual acuity remained unchanged 15 days after the procedure, as did the visual field defect.

The following factors might explain the rapid visual loss and visual field loss and the reason for poor visual outcome. First, poor initial visual acuity. Second, optic canal bony dehiscence was identified from CT scan. That may result optic nerve dehiscence. Third, content of the posterior ethmoid mucocele was purulent and mucoid. Direct spread of mucocele-initiated inflammation into the optic nerve sheath might occurred. Fukuda et al. suggested that ischemia and inflammation induce an acute onset or deterioration of ophthalmologic signs and symptoms, which may indicate a poor prognosis [5].

If the patient has optic canal bony dehiscence or purulent content in mucocele, it might be the poor prognostic factor. Furthermore posterior ethmoid cell prevalence in Korean population was revealed 47.3% in the study using computed tomography (CT) [6]. Posterior ethmoid cell prevalence is higher in Korean population than in other countries, so if any Korean patients who presented with sudden deterioration of visual acuity or visual field, or periocular pain, conducting imaging study is considerable to distinguish optic neuropathy caused by mucocele.

The publication and comparative analysis of additional cases of mucocele-associated optic neuropathy are necessary to establish an effective management.

## Data Availability

The datasets used and/or analysed during the current study available from the corresponding author on reasonable request.
